# The relationship between occupational stressors and insomnia in hospital nurses: The mediating role of psychological capital

**DOI:** 10.3389/fpsyg.2022.1070809

**Published:** 2023-02-14

**Authors:** Mei-Fang Wang, Pei Shao, Chao Wu, Lin-yuan Zhang, Lan-fang Zhang, Juan Liang, Juan Du

**Affiliations:** ^1^Department of Nursing, Xi’an Jiaotong University City College, Xi’an, China; ^2^School of Nursing, The Fourth Military Medical University, Xi’an, China; ^3^Department of Pediatrics, The First Affiliated Hospital of Air Force Military Medical University, Xi’an, China

**Keywords:** insomnia, psychological capital, occupational stress, job demand-control-support model, hospital nurses

## Abstract

**Background:**

Nurses have a high incidence of insomnia. Insomnia not only damages the physical and mental health of nurses, but also reduces their productivity and quality of care, ultimately affecting patient care. Over the past 30 years, a large number of epidemiological surveys have shown that insomnia in nurses is associated with occupational stress. As an external feature of the role of a nurse, occupational stress is difficult to alter in a short period of time. Therefore, it is necessary to discuss the complex mediating variables in the relationship between occupational stress and insomnia in nurses in order to find different ideas to address the problem of insomnia caused by occupational stress. Psychological capital, the positive psychological strength of an individual, has been widely used in previous reports as a mediating variable between occupational stress and adverse psychological problems.

**Objective:**

This study aimed to explore the mediating effect of psychological capital on occupational stressors and insomnia among Chinese nurses.

**Methods:**

The Strengthening the Reporting of Observational Studies in Epidemiology statement was referred to conduct the study. A cross-sectional stratified sampling method was used to recruit 720 participants from a tertiary hospital in Jinan, Shandong province, located in the east of China, from June to August 2019. Questionnaires were used to obtain data on demographic variables, psychological capital, occupational stressors, and insomnia.

**Results:**

The study findings revealed that work settings [department (*F* = 3.08, *p* = 0.006), working hours per week (*t* = −2.03, *p* = 0.043) and shift work (*t* = 3.66, *p* < 0.001)], decision latitude (*r* = −0.25, *p* < 0.001), psychological job demand (*r* = 0.15, *p* < 0.001), social support (*r* = −0.31, *p* < 0.001), and psychological capital (*r* = −0.40, *p* < 0.001) were differentially associated with insomnia experiences. This cross-sectional survey showed that psychological capital has significant mediation effects on the relationship between occupational stressors and insomnia. In the model of decision latitude - psychological capital - insomnia, the mediating effect was-0.04 (95%CI: −0.07 ~ −0.02), accounting for 50.0% of the total effect; In the model of job demands – psychological capital – insomnia, the mediating effect was 0.03 (95%CI: 0.01 ~ 0.06), accounting for 25.0% of the total effect; In the model of social support - psychological capital - insomnia, the mediating effect was −0.11 (95%CI: −0.16 ~ −0.07), accounting for 39.0% of the total effect.

**Conclusion:**

Psychological capital not only had a direct effect on both occupational stressors and insomnia, but also played mediating roles in relationship between occupational stressors and insomnia. It has been suggested that nurses themselves and nursing managers should improve the psychological capital of nurses by various means to alleviate the effects of occupational stress on nurses’ insomnia.

## 1. Introduction

Insomnia is a common sleep disorder in population. More than a third of the world’s population suffers from insomnia (Brownlow et al., 2020; Serrano-Ripoll et al., 2021). Nurses are prone to many sleep problems due to the nature of their profession (Zhan et al., 2020; Oteir et al., 2022). Insomnia prevalence in nurses was reported to be approximately 30 to 65.4% worldwide, and 37–63.9% in China (Pappa et al., 2020; Zhan et al., 2020; Lyu et al., 2022). Insomnia impairs individuals’ cognitive, physical, and emotional functions, greatly reducing the quality of life and adversely affecting health (Fernandez-Mendoza et al., 2015; Li et al., 2015; Brownlow et al., 2020). For example, studies have shown that a lack of sleep negatively affects the immune system and metabolism, increasing the risks of mental disorders, depression, coronary heart disease, stroke, morbid obesity, etc. (Troxel et al., 2010; Fernandez-Mendoza et al., 2015; Li et al., 2015). Additionally, insomnia can reduce the attention span and weaken the memory of nurses, which can lead to emotional instability as well as other psychological disorders. Furthermore, insomnia affects the efficiency and quality of nursing work, leading to nursing errors, accidents, and disputes (Dong et al., 2017). The economic loss caused by insomnia has been increasing in recent years, including increased medical and health care expenses (Kessler et al., 2011; Sarsour et al., 2011). Therefore, insomnia is one of the most concerning health problems among nursing community.

Occupational stress is a crucial factor affecting insomnia. In the past 30 years, numerous epidemiological studies have investigated the relationship between insomnia and occupational stress (Yang et al., 2018) and have reported that occupational stress is related to poor sleep quality. Studies have shown that nurses generally have high occupational stress. In health services, the complexity of work has grown rapidly over the past few decades as medical workers have had to learn a myriad of different and complex things. This leads to increased job demands and job stress for nurses in healthcare institutions such as hospitals, where rapid growth in job demand and increased job stress can lead to health problems (Kessler et al., 2011; Sarsour et al., 2011). Research has shown that job stress was a key issue for the health and safety of nurses. Furthermore, studies have documented that risk factors for occupational stress include high psychological job demand (PSD), low decision latitude (DL)- low range of decision-making freedom (control) available to the worker facing those demand, and low social support (SS; van der Molen et al., 2020). The influence of these risk factors in occupational stress is considered in Karasek’s job demand-control-support model (Karasek, 1990). Karasek’s model assumes that occupational stress is the result of high PSD, low SS, and low DL, which are psychosocial work stressors (Jansson and Linton, 2006; Jansson-Fröjmark et al., 2007). In addition, occupational stress caused by high PSD has been reported to lead to physical and psychological problems among working populations in different societies (Jalilian et al., 2019; van der Molen et al., 2020). Low SS and low DL predicted an increase in insomnia, and both confirmed the role of high PSD (Jansson and Linton, 2006; Jansson-Fröjmark et al., 2007; Portela et al., 2015; Saijo et al., 2015). Nurses must maintain high concentration because their job profile is linked to patient safety and satisfaction. Nurses must pay special attention to serious, careful nursing operations to ensure the physical safety of patients, as well as to their emotional expression when communicating directly with patients (Yang et al., 2018; Garefelt et al., 2020). Nurses are thus a vulnerable and high-risk population for occupational stress. Previous research has found that occupational stressors have an impact on insomnia both directly and indirectly (Jansson and Linton, 2006; Jansson-Fröjmark et al., 2007; Portela et al., 2015; Saijo et al., 2015). Portela showed that occupational stress was a major risk factor for insomnia in nurses (Zhan et al., 2020). At present, approximately 26.8–69.7% of nurses have different degrees of sleep problems due to occupational stress (Garefelt et al., 2020; Zhan et al., 2020; Oteir et al., 2022). However, occupational stress caused by external factors related to the nature of the nursing profession cannot be changed in the short term. Therefore, the complex mediating variables in the relationship between occupational stress and insomnia in nurses must be explored to reduce the effect of occupational stress on sleep quality in nurses through the effective intervention of the mediating variables.

Previous studies on the factors influencing insomnia have focused on demographic or behavioral factors, such as gender, age, physical or mental problems (Zhan et al., 2020; Oteir et al., 2022), alcohol consumption frequency, and eating habits (Thakkar et al., 2015), and have often ignored positive psychological qualities of an individual and their potential effect on sleep quality. Therefore, the relationship between occupational stress and insomnia in nurses must be explored from the perspective of positive psychology to find novel interventions for insomnia prevention in nurses.

Positive personal traits effectively combat occupational stress and insomnia (Huang et al., 2016). Psychological capital (PsyCap) refers to the positive psychological state of individual development, and the four elements that constitute PsyCap are self-efficacy, hope, optimism, and resilience (Sun et al., 2012; Kwok et al., 2015). PsyCap is characterized by (1) having confidence (self-efficacy); (2) making positive attributions regarding present and future success (optimism); (3) pursuing a goal persistently and, if necessary, redirecting the path to the goal to achieve success (hope); and (4) maintaining equanimity, bouncing back, and even surpassing to succeed when faced with problems and adversity (resilience). During challenging events or in a new environment, individuals with a high PsyCap have the confidence to adapt; they can set reasonable goals and make positive plans, have expectations, have strong motivation, and be hopeful of achieving the goals; they are optimistic, attributing their failures to unstable external factors and their successes to internal factors; and they are not willing to give up easily even when they encounter setbacks and failures and can persist to the end.

The positive role of PsyCap in different occupations has been explored (Sun et al., 2012; Kwok et al., 2015). Occupational stress has been found to be negatively related to psychological capital, and it has been shown that occupational stress can lower a person’s PsyCap (Khalid et al., 2020; Shah et al., 2021). Both SS and DL predicted an increase in PsyCap, which contradicted the role of PSD (Choi and Yoon, 2017; López-Núñez et al., 2020; Xi et al., 2020; Xia et al., 2022). In addition, the role of PsyCap as a mediator has attracted the attention of many researchers in various fields, including medicine and education. For example, studies have shown that PsyCap played a mediating role in the relationship between occupational stress and negative emotions, depression tendency, job burnout, job performance, job satisfaction, and mental health (Peng et al., 2019; Khalid et al., 2020; Shah et al., 2021). Studies have shown that high occupational stress was a risk factor for a low level of PsyCap (Shah et al., 2021). Therefore, through the enhancement of PsyCap and its dimensions, the occupational stress of nurses can be reduced to improve their physical and mental health. PsyCap was found to be significantly negatively correlated with sleep disorders in rural primary and secondary school teachers by Huang et al. (2016). Peng et al. showed that PsyCap influences both occupational stress and sleep quality (Peng et al., 2019). Studies have shown that overly stressful days were often followed by difficulty falling asleep and staying asleep, as well as increased stress and occupational tension (Åkerstedt et al., 2012; Halonen et al., 2017). However, decreased PsyCap caused by occupational stress would further increase the possibility of insomnia in nurses. Although the relationship between occupational stressors and insomnia, or between PsyCap and insomnia, has been established in a variety of professions (Jansson and Linton, 2006; Portela et al., 2015; Huang et al., 2016; Yang et al., 2018), the mediating role of PsyCap between occupational stressors and insomnia based on Karasek’s demand-control-support model has not been studied in nurses to our knowledge. To address this gap and find intermediate factors that counter the effects of occupational stress on insomnia, interventions and training on intermediate factors at a later stage can lead to new ideas for the prevention and treatment of insomnia due to occupational stress, this study focuses on a Chinese population and investigates the potential mediating effect of PsyCap on the posited association between occupational stressors and insomnia. Therefore, we hypothesized that PsyCap might have mediating effects on the relationship between nurses’ occupational stressors based on Karasek’s demand-control-support model and insomnia. This study was therefore conducted to investigate the mediating effect of PsyCap on the relationship between occupational stress dimensions and insomnia among Chinese nurses based on Karasek’s demand-control-support model.

## 2. Methods

The Strengthening the Reporting of Observational Studies in Epidemiology statement (Cuschieri, 2019) was referred to conduct this study.

### 2.1. Study design and participants

A cross-sectional stratified sampling method was used to recruit participants from a tertiary hospital in Jinan, Shandong province, located in the east of China, from June 2019 to August 2019. In total, 720 nurses were investigated in the survey, among which 119 were men and 601 were women. Clinical registered nurses performing clinical nursing work for >1 year were recruited for the study after obtaining their informed consent. The exclusion criteria of the study were (1) nurses on sick leave or maternity leave during the study period; (2) advanced students or on-the-job logistics personnel; and (3) nurses who recently experienced any traumatic event in their lives. After institutional review board approval was obtained, anonymous questionnaires were sent out to nurses who met the inclusion and exclusion criteria by uniformly trained investigators, and standardized explanations were provided if necessary.

### 2.2. Sample size calculation

The sample size was calculated using the descriptive study simple random sampling calculation formula $N=\frac{Z_{1-\alpha /2}^{2}\left(1-P\right)}{\varepsilon^{2}P}$ (Sun and Xu, 2014). According to the literature, the average prevalence rate of insomnia in nurses was 40.0% (Pappa et al., 2020; Zhan et al., 2020; Lyu et al., 2022; Oteir et al., 2022). With 95% confidence and 10% allowable error, *N* = 576 was calculated. Considering a dropout rate of 20%, the sample size was determined to be 720.

### 2.3. Sampling procedures

First, the sample of the survey was 720 nurses; the ratio of the sample size to the number of individuals in the population was determined to be 720:1320 = 6:11; then, the sampling ratio was used to calculate the number of nurses to be selected in the departments of internal, external, obstetrics and gynecology, pediatrics, emergency, operating room and ICU, which were 155, 218, 65, 46, 42, 84, and 110, respectively; the simple random sampling method was used to randomly select the corresponding number of samples from the above departments for analysis (Take a department as one unit, for example, the number of nurse satisfying the inclusion criteria in this department was N, and the number of nurse to be selected according to the sampling ratio was M, then numbered the N nurses in turn, wrote the number on the number label, placed the number label in an opaque container and stirred evenly, one number was drawn from it each time, and did not put back to draw M times continuously. Individuals corresponding to the m-number labels were chosen as the department’s investigation objects).

### 2.4. Data collection

1. Before the investigation, the investigators issued notification letters to all the investigated subjects face to face to ensure their right to know, and explained the purpose and significance of the investigation and the work needed to cooperate with the investigated departments, so as to obtain the active support and cooperation of the investigated subjects and departments.
2. During the investigation, the investigators obtained the strong cooperation of the head nurse of the department, special locations and times were arranged, and field investigators distributed questionnaires uniformly to the respondents face to face and explained the contents of the investigation and the main items to the respondents with uniform instructions. Concentrated filling was carried out and questionnaires were collected on the spot. During the questionnaire recovery process, if a missing item is found in the questionnaire, the respondent should promptly make up the missing item.

In this study, 691 completed questionnaires were returned (response rate, 96.0%). The data from 658 participants (91.4% of 720) were analyzed in this study after some questionnaires with missing information were removed.

### 2.5. Measures

A demographic questionnaire, Job Content Questionnaire (JCQ), Psychological Capital Questionnaire (PCQ-24), and Athens Insomnia Scale (AIS) were used for data collection, and the average time provided for completion of the questionnaires was 10 min.

#### 2.5.1. Measurement of basic information

The demographic questionnaire included gender, age, educational background, department, working hours per week, and monthly income.

#### 2.5.2. Measurement of occupational stressors

A simplified Chinese version of JCQ, translated and developed by Li et al. (2004), was used to measure occupational stressors. The questionnaire consists of 22 items and three dimensions, namely DL, PSD, and SS, which consists of nine, five, and eight items, respectively. Items in the scales were scored using a 5-point Likert scale in which 1 indicated strongly disagree and 5 indicated strongly agree, and the score of each dimension was calculated according to Dr. Li’s questionnaire scoring method. Studies have shown that this scale has good reliability and validity (Li et al., 2004). In this study, Cronbach’s α for DL, PSD, and SS subscales were 0.836, 0.861, and 0.931, respectively, for Chinese nurses.

#### 2.5.3. Measurement of PsyCap

The PsyCap of nurses was measured using the PCQ-24 (Luthans et al., 2006). It has four dimensions, namely self-efficacy, hope, resilience, optimism, with six items for each dimension, and each item was scored on a 5-point Likert scale from 1 (strongly disagree) to 5 (strongly agree). The sum of all items can be considered the result of the PCQ-24, and the total score was used to measure PsyCap. The higher the score, the higher the PsyCap level of the participant. Domestic studies have shown that the PCQ-24 has good reliability and validity for the cultural background of China (Sun et al., 2012; Huang et al., 2016). In this study, Cronbach’s α for the total scale was 0.96, showing good reliability.

#### 2.5.4. Measurement of insomnia

Insomnia of nurses was measured using the Athens Insomnia Scale (AIS; Soldatos et al., 2000). The AIS consists of eight items: Each item was rated 0, 1, 2, or 3 from none to severe, and the range for insomnia was 0–24, with higher scores indicating poorer sleep quality. Due to its accurate self-measurement results and convenient use, this scale is widely used in clinical practice and has been recognized by the international medical community as a standard scale for insomnia evaluation (Pappa et al., 2020). In this study, Cronbach’s α was 0.89, showing good reliability.

### 2.6. Statistics

Excel 2013 was used for the double entry of data, and SPSS 22.0 software was used for the statistical analysis. Kolmogorov–Smirnov (K-S) single sample test and P–P plot were used to test the Gaussian distribution of the data. The results showed that the K-S test *p* > 0.05, and the data points in the P–P plot coincided with the theoretical line. Thus, the data obeyed the Gaussian distribution. The descriptive analysis is presented as counts (*n*) and percentages (%); independent-sample *t*-test and one-way analysis of variance were used to compare the demographic data. The Least Significant Difference method was used for post-comparison when the variances of pair comparisons between multiple groups were homogeneous. Pearson correlation analyses of the three variables (occupational stress, PsyCap, and insomnia) were performed. Model 4 in-process version 3.4 developed by Hayes (2018) was used to test the mediating effect of PsyCap on the relationship between occupational stress and insomnia, in which all types of variables were represented as dummy variables and statistically significant confounding factors in univariate analysis were considered covariates in the analysis. This approach was based on ordinary least-squares regression and the bootstrap method with 5,000 bootstrap bias-corrected 95% confidence intervals (BC CI) to be used for mediation analyses. The significance test level was *p* < 0.05.

### 2.7. Ethics statement

This study was approved by the Ethics Committee of Qianfoshan Hospital (Grant number: 2022S007). The attributes, benefits, uses, and disadvantageous effects of the study were explained to all participants, and their informed consent was also obtained.

## 3. Results

### 3.1. Demographic information of the respondents

Table 1 presents the demographics and work-related variables of the sample. Among the 658 nurses studied, 109 were men, and 549 were women; 71.9% were ≤30 years old, 20.0% were 30–40 years old, and 8.1% were >40 years old; 78.0% had a bachelor’s degree, and 3.6% had a master’s degree; 36.3% worked for >40 h per week, and 74.3% worked shifts.

**Table 1:** Demographic statistics and the influence of demographic information and life behavior factors on insomnia.

Variables	Number (*N*)	Percentage (%)	Insomnia		*T*/*F*	*p*	*Post hoc* test/Mean deviation	
			Mean	SD				
Gender					−0.00	>0.999		
Men	109	16.6	7.70	5.05				
Woman	549	83.4	7.70	4.54				
Age(years)					0.37	0.690		
① ≤30	473	71.9	7.62	4.42				
② 30–40	132	20.0	8.01	5.38				
③ >40	53	8.1	7.62	4.40				
Education					1.41	0.244		
① Junior college and below	121	18.4	7.48	4.05				
② College	513	78.0	7.81	4.71				
③ Postgraduate	24	3.6	6.29	5.38				
Departments					3.08	0.006		
① Internal Medicine	109	16.6	7.72	4.38			①>③	1.49^*^
② Surgery	213	32.4	7.80	4.67			②>③	1.57^*^
③ Gynaecology and Obstetrics	61	9.3	6.23	4.66			⑤>③	2.85^*^
④ Pediatrics	42	6.3	7.07	5.00			⑤>④	2.01^*^
⑤ Emergency	39	5.9	9.08	4.52			⑤>⑥	2.21^*^
⑥ Operating room	84	12.8	6.87	3.75			⑦>③	2.44^*^
⑦ Intensive care units (ICUs)	110	16.7	8.67	4.96			⑦>⑥	1.80^*^
Weekly working hours (hours)					−2.03	0.043		
≤40	419	63.7	7.42	4.39				
>40	239	36.3	8.18	4.98				
Monthly income (RMB)					1.59	0.205		
≤5,000	213	32.4	7.40	4.60				
5,000–8,000	339	51.5	7.67	4.61				
>8,000	106	16.1	8.38	4.66				
Shift work					3.66	<0.001		
Yes	489	74.3	8.08	4.73				
No	169	25.7	6.59	4.12				

SD is the abbreviation of standard deviation.

* *p* < 0.05.

### 3.2. Univariate analysis of the influence of demographic factors on insomnia

The working department (*F* = 3.08, *p* = 0.006), working hours per week (*t* = −2.03, *p* = 0.043) and shift work (*t* = 3.66, *p <* 0.001) had significant effects on insomnia (Table 1). Insomnia scores of nurses in internal medicine, surgery, emergency departments, and intensive care units (ICUs) were higher than those of obstetrics and gynecology nurses. Furthermore, the insomnia scores of nurses in emergency departments and ICUs were higher than those of operating room nurses. Additionally, the insomnia scores of nurses in emergency departments were higher than those of pediatric nurses. Nurses who worked >40 h a week had higher scores of insomnia than those who worked ≤40 h a week. The insomnia score of shift nurses was higher than that of non-shift nurses.

### 3.3. Bivariate correlation analysis

Insomnia was positively correlated with PSD (*r* = 0.15, *p* < 0.001) and negatively correlated with DL (*r* = −0.25, *p* < 0.001), SS (*r* = −0.31, *p* < 0.001), and PsyCap (*r* = −0.40, *p* < 0.001; Table 2). PsyCap was significantly positively correlated with DL (*r* = 0.33, *p* < 0.001) and SS (*r* = 0.39, *p* < 0.001) and significantly negatively correlated with PSD (*r* = −0.11, *p* < 0.001).

**Table 2:** Bivariate correlation analysis.

	Occupational stressors			PsyCap	Insomnia
	DL	PSD	SS		
DL	1				
PSD	−0.06	1			
SS	0.42^**^	−0.13^**^	1		
PsyCap	0.33^**^	−0.11^**^	0.39^**^	1	
Insomnia	−0.25^**^	0.15^**^	−0.31^**^	−0.40^**^	1
Mean	80.27	41.37	33.17	101.59	7.70
Standard deviation	13.23	5.88	4.97	17.02	4.62

DL stands for decision latitude; PSD stands for psychological job demand; PsyCap stands for Psychological Capital; SS stands for social support.

** *p* < 0.01.

### 3.4. Relationship between occupational stressors and insomnia: Mediation model tests of PsyCap

**Figure 1:**
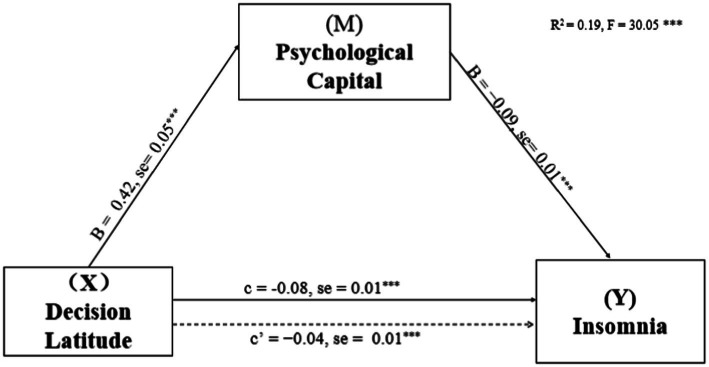
Mediation of Psychological capital in the relationship between decision latitude and insomnia symptoms with non-standardized beta values and standard error. ***p < 0.001.

**Figure 2:**
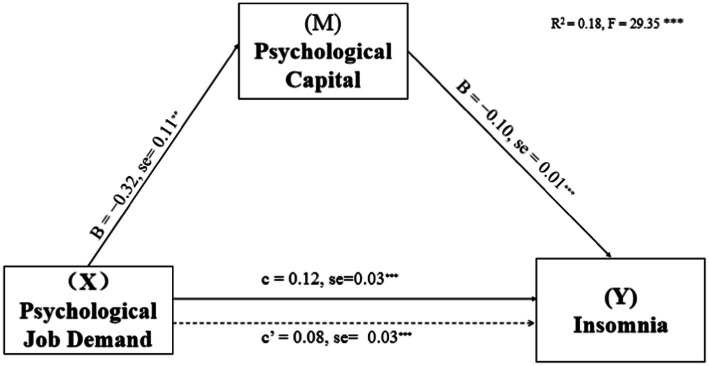
Mediation of Psychological capital in the relationship between psychological job demand and insomnia symptoms with non-standardized beta values and standard error. **p < 0.01, ***p < 0.001.

**Figure 3:**
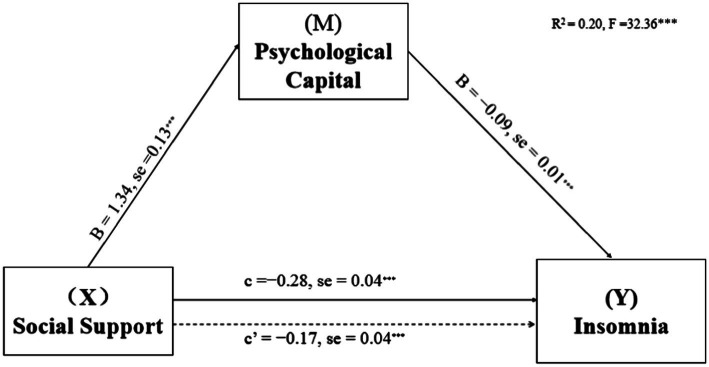
Mediation of Psychological capital in the relationship between social support and insomnia symptoms with non-standardized beta values and standard error. ***p < 0.01.

Based on work department working hours per week and shift work, the mediating effects of PsyCap on the relationship of DL, PSD, and SS with insomnia were investigated. PsyCap played a mediating role in their models (see Figures 1–3; Table 3). As shown in Figures 1–3, DL, PSD, and SS had significant predictive effects on insomnia (*c* = −0.08, *t* = −6.35, *p* < 0.001; *c* = 0.12, *t* = 3.83, *p* < 0.001; and *c* = −0.28, *t* = −8.01, *p* < 0.001, respectively), and when PsyCap was included as the mediating variable, the direct predictive effects of DL, PSD, and SS on insomnia were still significant (*c*’ = −0.04, *t* = −3.41, *p* = 0.001; *c*’ = 0.08, *t* = 2.95 *p* = 0.003; and *c*’ = −0.17, *t* = −4.60, *p* < 0.001, respectively). DL, PSD, and SS had significant predictive effects on PsyCap (B = 0.42, *t* = 8.88, *p* < 0.001; B = −0.32, *t* = −2.88, *p* = 0.004; and B = 1.34, *t* = 10.70, *p* < 0.001, respectively). Moreover, the negative predictive effect of PsyCap on insomnia was significant in the models with independent variables, including DL, PSD, and SS (B = −0.09, *t* = −8.97, *p* < 0.001; B = −0.10, *t* = −10.26, *p* < 0.001; and B = −0.09, *t* = −8.14, *p* < 0.001, respectively). PsyCap played mediating roles in the DL-PsyCap-insomnia model, PSD-PsyCap-insomnia model, and SS-PsyCap-insomnia model. In addition, the upper and lower limits of the 95% confidence intervals of the direct effects of DL, PSD, and SS on insomnia and the mediating effects of PsyCap did not contain 0 (Table 3), indicating that DL, PSD, and SS could not directly predict insomnia. Moreover, they could predict insomnia through the mediating role of PsyCap. In the DL-PsyCap-insomnia model, the mediating effect (−0.04) and direct effect (−0.04) accounted for 50.0% and 50.0% of the total effect (−0.08), respectively. In the PSD-PsyCap-insomnia model, the mediating effect (0.04) and direct effect (0.08) accounted for 25.0 and 75.0% of the total effect (0.12), respectively. In the SS-PsyCap-insomnia model, the mediating effect (−0.11) and direct effect (−0.17) accounted for 39.29% and 60.71% of the total effect (−0.28), respectively.

**Table 3:** The total effect, direct effect, and mediating effect of occupational stressors – psychological capital – insomnia.

M:PsyCap						
Model	Effect	Effect size	Boot SE	Lower level 95% CI	Upper level	Relative effect value
					95% CI	
Y:Insomnia	Mediating effect	−0.04	0.01	−0.07	−0.02	50.00%
X:DL	Direct effect	−0.04	0.01	−0.07	−0.02	50.00%
M:PsyCap	Total effect	−0.08	0.01	−0.11	−0.06	
Y:Insomnia	Mediating effect	0.04	0.01	0.01	0.06	25.00%
X:PSD	Direct effect	0.08	0.03	0.03	0.14	75.00%
M:PsyCap	Total effect	0.12	0.03	0.06	0.17	
Y:Insomnia	Mediating effect	−0.11	0.02	−0.16	−0.07	39.29%
X:SS	Direct effect	−0.17	0.04	−0.24	−0.1	60.71%
M:PsyCap	Total effect	−0.28	0.04	−0.35	−0.21	

Three dimensions of occupational stress: DL, PSD, and SS. DL stands for decision latitude; PSD stands for psychological job demand; SS stands for social support. PsyCap stands for Psychological Capital. The Bootstrap results are based on 5,000 Bootstrap samples.

## 4. Discussion

Previous studies on the relationship between nurses’ occupational stressors and insomnia have focused on the direct effect, but research on its internal mediating mechanism has been lacking. This study used PsyCap to explore the mechanism by which nurses’ occupational stressors based on Karasek’s model is related to insomnia, which is an expansion of previous studies, providing a deeper understanding of their findings. The results showed that the occupational stressors of nurses could directly affect insomnia, and it could also indirectly affect insomnia through PsyCap. PsyCap plays mediating roles between occupational stressors and insomnia.

### 4.1. Analysis of factors influencing insomnia in nurses

This study revealed that the main factors influencing insomnia are the work department, working hours per week, shift work, DL, PSD, SS, and PsyCap. Nurses in surgery, in emergency departments, in ICUs, working >40 h a week, on shifts, with high PSD, with low DL, with low SS, and with low PsyCap had high scores of insomnia.

In the surgical working environment, patients are received in critical condition, and nursing work is characterized by numerous and high-intensity tasks, which causes surgical nurses long-term stress and makes them prone to insomnia. Emergency department nurses are involved in the long-term treatment of acute and severe patients, and the tension is not relieved. In addition, emergency departments have a high incidence of medical disputes, and nurses involved often encounter patients with injury and traffic accident trauma, causing undesirable stimulation, and they have to respond to emergent public health events at any time, causing immense stress mentally and physically. These factors decrease their sleep quality. In ICUs, patients with acute and critical illnesses are treated, and ICU patients are characterized by a long age span, severe disease, rapid changes in the disease state, and a high mortality rate. In addition, ICU nursing operation techniques are relatively difficult, leading to long-term, high-intensity, and high-pressure working environments for ICU nurses, which seriously affect their physical and mental health and sleep quality (Aroca et al., 2013).

This study found that nurses who worked >40 h a week scored higher in insomnia, which was similar to the results of some studies (Virtanen et al., 2009; Nakashima et al., 2011). However, studies have reported varying results regarding whether working overtime is an independent risk factor for insomnia. Some studies (Basner et al., 2007; Virtanen et al., 2009; Nakashima et al., 2011) have shown that working >40 h per week is a risk factor for insomnia without considering mental health. However, some studies have implied that with good psychological health, average weekly working duration of >40 h was unrelated to insomnia, but poor mental health combined with an average weekly working duration of ≤40 h was found to be a risk factor for insomnia (Akerstedt et al., 2002a,b), which indicated a potentially strong interaction between working duration of >40 h per week and mental health status on insomnia. The population with good mental health status has relatively good psychological adjustment ability, and overtime working does not have a qualitative effect on sleep in this case. Some scholars believe that poor mental health is already a risk factor for insomnia, and overtime work would increase insomnia risk (Rahmani et al., 2020). Therefore, further studies are needed to confirm whether overtime work is an independent risk factor for insomnia.

Table 2 and the correlation between occupational stressors and insomnia suggest that the study results are reasonable because high PSD increases psychological pressure and can finally lead to insomnia. Table 2 demonstrates a negative relationship between DL and insomnia. These findings were in accordance with those of a previous study (Garefelt et al., 2020). This implies that if nurses have more decision control on a given task, they will be motivated to fulfill their responsibilities. Increasing nurses’ DL is good for nurses’ physical and mental health. Moreover, the current study findings showed a negative correlation between SS and insomnia. This indicates that insomnia is related to workplace conditions, such as coworker support and supervisor support. Therefore, developing a friendly working environment and improving the support of leaders and colleagues can reduce insomnia occurrence in nurses (Saijo et al., 2015).

### 4.2. Mediating effect of PsyCap on occupational stressors and insomnia among nurses

In this study, PsyCap not only directly affected both occupational stressors and insomnia but also played a mediating role in the relationship between occupational stressors and insomnia. First, the study confirmed that a significant negative correlation exists between PsyCap and insomnia; that is, the higher the PsyCap level of nurses, the less prone they are to insomnia. Second, this study revealed that PsyCap plays a mediating role in their models, indicating that occupational stressors could predict nurses’ insomnia through the mediating role of PsyCap. The results supported the view of previous studies (Huang et al., 2016), that is, PsyCap, as an essential component of personal resources, affects individuals’ conscious control over their thoughts and actions (Shah et al., 2021). Individuals with higher PsyCap can deal with problems effectively, expect good results, recover quickly from setbacks, and face negative situations with a good attitude, which can buffer the adverse effects of occupational stress on individual sleep (Choi and Yoon, 2017).

Nurses’ DL and SS were significantly positively correlated with PsyCap. Improving nurses’ decision-making freedom and SS could improve their PsyCap and indirectly affect their sleep quality. This may be mainly because with the continuous increase of DL with PSD, the devotion of nurses to their work increases, and they have increased goals and self-confidence in their work, which improves their self-efficacy and hope (Portela et al., 2015; Saijo et al., 2015; Xia et al., 2022). Simultaneously, the improvement of DL will cause nurses to have positive emotions. When nurses are faced with difficult work, getting material help, and spiritual support from the hospital can improve their PsyCap (Portela et al., 2015; Wang et al., 2017; Shah et al., 2021). With the overall improvement of PsyCap in nurses, their insomnia will be alleviated and reduced eventually. Therefore, the higher the level of individual’s PsyCap, the more positive emotions experienced, and the lower the insomnia symptoms (Shah et al., 2021).

### 4.3. Implication

This study suggests that clinical nursing leaders should improve nurse decision-making freedom and reduce the psychological needs of nurses at work. At the same time, nurse leaders should become active social supporters of nurses. Hospital administrators should make every effort to improve nurses’ PsyCap to help nurses boost sleep quality when occupational stress is unavoidable. Nurses themselves should find ways to improve their psychological capital to combat the occupational stress of insomnia. When occupational stress, as the external characteristics brought by the role of nurses, is difficult to alter in a short period of time, it suggests finding ways to improve the psychological capital of nurses, so as to counter the influence of occupational stress on insomnia, to reduce the incidence of insomnia and promote the physical and mental health of nurses.

### 4.4. Limitations

This study has some limitations. First, this is a cross-sectional study, and hence, conclusions obtained through this study cannot be used to infer causality and can only provide a description of the current situation or of the interrelationship. Second, although the survey quality was controlled, the questionnaire was self-filled, and psychological questionnaires have strong subjectivity, resulting in a potential self-report bias. Finally, although the Job Demand-Control-Support Model is widely used in the world, it is seldom applied in China. Therefore, further studies are needed to verify the model of the theory and form a stable norm. Owing to these limitations, the conclusion obtained from this investigation can only be used as a description of the current situation, and its extrapolation is limited to a certain extent.

## 5. Conclusion

In this study, we first explored the factors influencing insomnia in Chinese nurses and then investigated the relationship between occupational stressors (based on the job demand-control-support model), PsyCap, and insomnia among Chinese nurses by using a mediation model. The results indicated that the work department, weekly working hours, shift work, DL, PSD, and SS were the main influencing factors of insomnia. PsyCap not only directly correlated both occupational stressors and insomnia but also played mediating roles in the relationship between occupational stressors and insomnia. Nursing managers must avoid allocating >40 h a week of work and arrange shifts properly to nurses and improve nurses’ decision-making freedom, reduce nurses’ psychological needs at work, and increase support for nurses to improve their PsyCap, thereby improving nurses’ sleep quality, promoting nurses’ health, and boost nurses’ nursing service quality. Furthermore, this study suggests that improving nurses’ PsyCap can potentially help nurses improve sleep quality when occupational stressors are unavoidable. For example, hospital administrators should develop a hospital staff assistance program, try to give nurses enough care, and try their best to meet the psychological needs of the nurses to increase confidence in nurses and make them hopeful regarding their life and work.

## Data availability statement

The datasets are available on reasonable request to the corresponding author.

## Author contributions

M-FW, JD, PS, and CW conceived and designed the research. JD, M-FW, PS, L-yZ, L-fZ, and JL performed the research. JD, M-FW, PS, L-yZ, and L-fZ performed the data analyses. M-FW, JD, PS, CW, and JL wrote the paper. All authors contributed to the article and approved the submitted version.

## Funding

This work was supported by the Shaanxi Province Education Science “13th Five-Year plan” Project (grant numbers SGH20Y1386) and New Flight Project of Air Force Military Medical University.

## Conflict of interest

The authors declare that the research was conducted in the absence of any commercial or financial relationships that could be construed as a potential conflict of interest.

## Publisher’s note

All claims expressed in this article are solely those of the authors and do not necessarily represent those of their affiliated organizations, or those of the publisher, the editors and the reviewers. Any product that may be evaluated in this article, or claim that may be made by its manufacturer, is not guaranteed or endorsed by the publisher.
